# AutoGluon-based interpretable machine learning for clinical risk stratification of retinopathy of prematurity in very preterm infants

**DOI:** 10.3389/fped.2026.1907714

**Published:** 2026-09-04

**Authors:** Siyu Chen, Shuyue Deng, Xinyi Liu, Wanting Xu, Wenbin Dong, Linxiao Wan, Lan Kang

**Affiliations:** Department of Neonatology, Children’s Medical Center, The Affiliated Hospital of Southwest Medical University, Luzhou, China

**Keywords:** AutoGluon, clinical risk stratification, interpretable machine learning, retinopathy of prematurity, SHAP, very preterm infants

## Abstract

**Introduction:**

Retinopathy of prematurity (ROP) remains an important cause of preventable visual impairment in preterm infants. Current screening relies primarily on gestational age and birth weight, which may not fully capture the heterogeneous postnatal clinical course of very preterm infants. This study aimed to develop and internally validate an interpretable machine-learning framework using routinely collected clinical variables for clinical-course-based in-hospital ROP risk stratification.

**Methods:**

A total of 236 infants were retrospectively included, comprising 106 infants with ROP and 130 infants with normal ROP screening findings during hospitalization. Fifteen routinely collected maternal and neonatal clinical variables were used in stratified five-fold internal validation. Within each fold, AutoGluon trained 13 individual candidate models and automatically constructed its built-in WeightedEnsemble_L2 from selected base-model predictions. Missing-data imputation and classification-threshold selection were performed within the training data of each fold. A separate 15-predictor LightGBM model was trained using the same clinical-variable framework and evaluated for TreeSHAP-based interpretation and local research-prototype implementation.

**Results:**

WeightedEnsemble_L2 was selected as the primary analytical model because of its overall discrimination and screening-oriented performance. It achieved an AUC of 0.823, sensitivity of 0.764, specificity of 0.677, positive predictive value of 0.659, and negative predictive value of 0.779. The Brier score was 0.171, with a calibration intercept of −0.095 and a calibration slope of 1.046. In the separately trained LightGBM model, mechanical ventilation duration, oxygen therapy duration, birth weight, bronchopulmonary dysplasia, length of hospital stay, and prematurity-related encephalopathy were the most influential predictors.

**Discussion:**

The proposed framework provides an interpretable, clinical-course-based approach to in-hospital ROP risk stratification by integrating routinely collected NICU data. It may enhance individualized risk assessment by incorporating clinical-course information beyond conventional static birth characteristics. Multicenter external and prospective validation is warranted to further evaluate its clinical applicability.

## 1 Introduction

Retinopathy of prematurity (ROP), a retinal vasoproliferative disease affecting preterm infants, remains one of the leading preventable causes of childhood blindness worldwide (1, 2). Advances in neonatal intensive care have substantially improved the survival of very preterm infants, while simultaneously increasing the population at risk for ROP, particularly in regions where screening, follow-up, and neonatal care resources are unevenly distributed. Timely ophthalmic screening and follow-up remain essential because severe visual impairment can often be prevented when clinically significant ROP is identified and treated promptly.

Current ROP screening strategies are still mainly based on GA and BW thresholds. In many clinical settings, infants with GA less than 32 weeks or BW less than 2,000 g are routinely referred for fundus screening (4, 5). This approach is simple and sensitive, but it does not fully reflect the heterogeneity of postnatal clinical courses among extremely preterm infants. Fundus examination also requires specialized imaging equipment, trained operators, and ophthalmologists experienced in neonatal retinal assessment. In addition, the procedure may be associated with transient adverse events, such as apnea, bradycardia, respiratory instability, infection risk, and gastrointestinal discomfort. Although the cost of screening and treatment is far lower than the lifelong burden caused by blindness (6), a more individualized risk stratification strategy may help clinicians identify infants who require closer surveillance while reducing unnecessary examinations in those at relatively low risk.

Recently, artificial intelligence (AI) and machine learning (ML) have been increasingly explored for ROP detection, classification, and risk stratification (7). Image-based deep-learning systems using color fundus photographs have shown promising performance in assisting diagnosis and staging (8). However, these systems depend on retinal imaging and dedicated acquisition workflows. Clinical-data-based approaches address a different use case by summarizing maternal, neonatal, respiratory, infectious, and metabolic information recorded during NICU care. Clinical-data-based models may provide a useful complement to existing screening strategies. Compared with models based only on GA and BW, models using routinely collected NICU data can incorporate respiratory support, oxygen exposure, infection status, metabolic indicators, and other aspects of the clinical course.

Therefore, this study aimed to develop and internally validate an interpretable machine-learning framework for clinical-course-based in-hospital ROP risk stratification in very preterm infants using routinely collected clinical variables. AutoGluon was used to train and compare 13 individual candidate models within a consistent internal-validation framework and to automatically construct its built-in WeightedEnsemble_L2 from the predictions of selected base learners. WeightedEnsemble_L2 served as the primary analytical model. Because LightGBM learners contributed substantially to the cross-validated ensemble and offered favorable model-specific interpretability, a standalone LightGBM model was trained separately using the same 15 predictors and preprocessing framework. This model was evaluated independently and used for TreeSHAP-based interpretation and local research-prototype implementation. An exploratory analysis further compared infants with and without treatment-indicated ROP.

## 2 Materials and methods

### 2.1 Study design and ethics

This study was a single-center retrospective cohort study of very preterm infants admitted to the Affiliated Hospital of Southwest Medical University between February 2019 and April 2025. After application of the prespecified eligibility criteria, 236 infants were included. The primary analysis was defined as retrospective clinical-course-based in-hospital risk stratification using cumulative information recorded during the index hospitalization. This retrospective study was conducted in accordance with reporting recommendations for ML-based clinical prediction research. The study protocol was reviewed and approved by the Medical Ethics Committee of the Affiliated Hospital of Southwest Medical University (approval number: KY2026057). Because this was a retrospective analysis of nonidentifiable clinical data, the requirement for informed consent was waived.

### 2.2 Data collection

Among the included infants, 106 had ROP documented during hospitalization, whereas 130 had normal ROP screening findings during hospitalization. Fifteen candidate predictors were prespecified before model development on the basis of conventional ROP screening criteria, previously reported ROP-related factors, clinical plausibility, and routine data availability. The predictors included birth weight, rural residence, lactate concentration, first whole-blood glucose concentration measured on postnatal day 1, gravidity, parity, in-hospital hyperglycemia, duration of oxygen therapy, length of hospital stay, duration of mechanical ventilation, bronchopulmonary dysplasia, neonatal pneumonia, prematurity-related encephalopathy, prenatal oligohydramnios, and clinical response at NICU admission (3). Because the study center serves a geographically diverse population in southwestern China, rural residence was prespecified as a candidate predictor to account for potential regional differences in perinatal care, healthcare accessibility, referral pathways, and neonatal management. Residence status was determined from the home address documented in the medical record. This variable was included for exploratory risk stratification.

### 2.3 Inclusion and exclusion criteria

The inclusion criteria were as follows: gestational age at birth greater than 23 weeks and less than 32 weeks, corresponding to 23 + 1 to 31 + 6 weeks, and a definite ROP screening result diagnosed by ophthalmologists at our hospital. Infants were excluded if they did not complete ROP screening, had severe congenital malformations, known chromosomal abnormalities, inherited metabolic diseases, or congenital ocular abnormalities such as congenital cataract, familial exudative vitreoretinopathy, congenital microphthalmia, or congenital microcornea.

### 2.4 Outcome definition

ROP screening and follow-up were performed in accordance with the 2014 Chinese guideline (9). ROP was diagnosed according to the International Classification of Retinopathy of Prematurity (ICROP) (10). The outcome was the presence of ROP documented during the inpatient screening period. Infants were classified into the documented ROP group (hereafter, the ROP group) if any stage of ROP was identified on at least one fundus examination during hospitalization. Infants with normal ROP screening findings during hospitalization were classified into the non-ROP group. Treatment-indicated ROP was defined as zone I ROP of any stage with plus disease, zone I stage 3 ROP without requiring plus disease, zone II stage 2 or 3 ROP with plus disease, or aggressive ROP requiring prompt treatment (9, 11). In accordance with ICROP3, incomplete retinal vascularization without a demarcation line, ridge, extraretinal neovascularization, or other ROP lesions was not considered ROP. Therefore, infants whose final inpatient examination showed incomplete vascularization but no ROP lesion were included in the non-ROP group. This classification was based on the available inpatient ROP screening findings.

### 2.5 Model development and validation

Model development and internal validation used stratified five-fold cross-validation. In each outer fold, the outer training data were further divided into an inner training subset and an inner validation subset at an 80:20 ratio. All data-dependent preprocessing, including iterative imputation, was fitted exclusively on the inner training subset. The Youden-index classification threshold was selected using predictions from the inner validation subset and was then applied unchanged to the outer test fold. The outer test fold did not influence imputation fitting, model training, model selection, or cutoff selection. AutoGluon version 1.2 was used to train 13 individual candidate models and to automatically construct one built-in WeightedEnsemble_L2 model. The individual candidate models were ExtraTreesEntr, ExtraTreesGini, RandomForestEntr, RandomForestGini, LightGBMXT, LightGBM, LightGBMLarge, CatBoost, XGBoost, NeuralNetFastAI, NeuralNetTorch, KNeighborsDist, and KNeighborsUnif. WeightedEnsemble_L2 combined predictions from selected first-level models, with the ensemble composition and weights learned by AutoGluon from the inner validation data using ROC-AUC as the optimization metric. Because model training was repeated within each outer fold, the selected base models and their weights could vary across folds. Random seed 42 was used throughout the locked analysis.

### 2.6 Model evaluation, interpretation, and prototype implementation

Model performance was evaluated from pooled outer-fold out-of-fold predictions using AUC, PR-AUC, sensitivity, specificity, PPV, NPV, accuracy, F1 score, Brier score, calibration intercept, calibration slope, and confusion-matrix counts. Ninety-five percent confidence intervals were estimated using 2,000 bootstrap resamples. Performance was reported for the 13 individual AutoGluon models and the built-in WeightedEnsemble_L2. WeightedEnsemble_L2 was selected as the primary analytical model because it provided strong discrimination together with a favorable balance of sensitivity and NPV.

For model interpretation and subsequent implementation, a separate 15-predictor LightGBM model was developed. This choice was motivated by the substantial contribution of LightGBM learners to the cross-validated ensemble and by the suitability of LightGBM for TreeSHAP interpretation and practical implementation. The model was trained and evaluated using the same fold-wise preprocessing and internal-validation framework. It was used for TreeSHAP analysis and local prototype development to support future clinical deployment. Global importance was summarized using mean absolute SHAP values, while signed SHAP values were used to describe the direction of feature contributions.

### 2.7 Statistical analysis

Statistical analyses were conducted using Python version 3.12.9 and R version 4.2.0. Baseline continuous and ordinal variables were summarized as mean ± standard deviation and compared using two-sided Mann–Whitney U tests. Categorical variables were presented as n/N (%) and compared using Pearson chi-square tests with Yates continuity correction. Analyses were based on available cases, and denominators therefore varied across variables.

The associations of ROP with bronchopulmonary dysplasia and prematurity-related encephalopathy were examined using available-case 2 × 2 contingency tables and reported as odds ratios and risk ratios with 95% confidence intervals. In the exploratory analysis of treatment-indicated ROP, continuous and ordinal variables were compared using Mann–Whitney U tests, categorical variables using Fisher's exact tests, and univariable associations were estimated as odds ratios with 95% confidence intervals. The Benjamini–Hochberg procedure was applied to control the false discovery rate across treatment-indication comparisons. All tests were two-sided, and *P* < 0.05 was considered statistically significant.

## 3 Results

### 3.1 Baseline characteristics

A total of 236 very preterm infants were included in the analysis. Of these, 106 were included in the ROP group, whereas 130 were included in the non-ROP group (Table 1). Across the 15 candidate predictors, 331 of 3,540 values were missing (9.4%), and 58 infants (24.6%) had complete data for all predictors. Detailed baseline characteristics and available-case denominators are presented in Supplementary Table S1, and missing-data characteristics and imputation diagnostics are summarized in Supplementary Supplementary Table S2.

**Table 1:** Baseline characteristics of the cohort by documented ROP status.

Variable	Total (*n* = 236)	Non-ROP (*n* = 130)	ROP (*n* = 106)	Statistic	*P* value
BW	1.25 ± 0.24	1.34 ± 0.21	1.15 ± 0.23	U = 10184.00	<0.0001
Lac	2.77 ± 1.88	2.91 ± 1.89	2.61 ± 1.86	U = 3124.50	0.3749
Blood_Glucose	5.17 ± 2.78	4.82 ± 2.56	5.61 ± 3.00	U = 5660.50	0.0406
Hospital_Days	49.27 ± 19.03	41.47 ± 13.02	58.84 ± 20.84	U = 3125.50	<0.0001
Ventilator_Days	23.50 ± 14.06	17.13 ± 9.51	31.39 ± 14.79	U = 2596.50	<0.0001
Oxygen_Days	38.55 ± 22.14	28.68 ± 15.44	50.77 ± 23.13	U = 2901.50	<0.0001
G	2.63 ± 1.59	2.67 ± 1.71	2.58 ± 1.45	U = 6866.00	0.9630
P	1.49 ± 0.97	1.44 ± 0.97	1.56 ± 0.98	U = 6394.00	0.3098
*Rural*				*χ*^2^ = 0.01	0.9097
Rural = 1	124/235 (52.8%)	69/129 (53.5%)	55/106 (51.9%)		
Rural = 0	111/235 (47.2%)	60/129 (46.5%)	51/106 (48.1%)		
*Hyperglycemia*				χ^2^ = 5.12	0.0237
Hyperglycemia = 1	132/207 (63.8%)	67/118 (56.8%)	65/89 (73.0%)		
Hyperglycemia = 0	75/207 (36.2%)	51/118 (43.2%)	24/89 (27.0%)		
*BPD*				χ^2^ = 32.04	<0.0001
BPD = 1	121/170 (71.2%)	44/86 (51.2%)	77/84 (91.7%)		
BPD = 0	49/170 (28.8%)	42/86 (48.8%)	7/84 (8.3%)		
*Pneumonia*				χ^2^ = 19.21	<0.0001
Pneumonia = 1	137/182 (75.3%)	56/92 (60.9%)	81/90 (90.0%)		
Pneumonia = 0	45/182 (24.7%)	36/92 (39.1%)	9/90 (10.0%)		
*Premature_Encephalopathy*				χ^2^ = 5.87	0.0154
Premature_Encephalopathy = 1	87/151 (57.6%)	40/83 (48.2%)	47/68 (69.1%)		
Premature_Encephalopathy = 0	64/151 (42.4%)	43/83 (51.8%)	21/68 (30.9%)		
*Oligo*				χ^2^ = 0.92	0.3384
Oligo = 1	23/236 (9.7%)	10/130 (7.7%)	13/106 (12.3%)		
Oligo = 0	213/236 (90.3%)	120/130 (92.3%)	93/106 (87.7%)		
*Reaction*				χ^2^ = 0.19	0.6618
Reaction = 1	193/231 (83.5%)	107/126 (84.9%)	86/105 (81.9%)		
Reaction = 0	38/231 (16.5%)	19/126 (15.1%)	19/105 (18.1%)		

Data are presented as mean ± SD for continuous and ordinal variables and as n/N (%) for categorical variables.

Continuous and ordinal variables were compared using two-sided Mann–Whitney U tests. Binary variables were compared using Pearson χ² tests with Yates continuity correction.

Compared with the non-ROP group, infants in the ROP group had a lower birth weight (median, 1.12 vs. 1.35 kg; *P* < 0.001), a higher first whole-blood glucose concentration on postnatal day 1 (5.45 vs. 4.30 mmol/L; *P* = 0.041), and longer durations of oxygen therapy (50 vs. 26 days), hospitalization (56.5 vs. 41 days), and mechanical ventilation (28 vs. 16 days; all *P* < 0.001). Hyperglycemia, bronchopulmonary dysplasia (BPD), pneumonia, and prematurity-related encephalopathy were also more frequent in the ROP group (all *P* ≤ 0.019).

**Figure 1:**
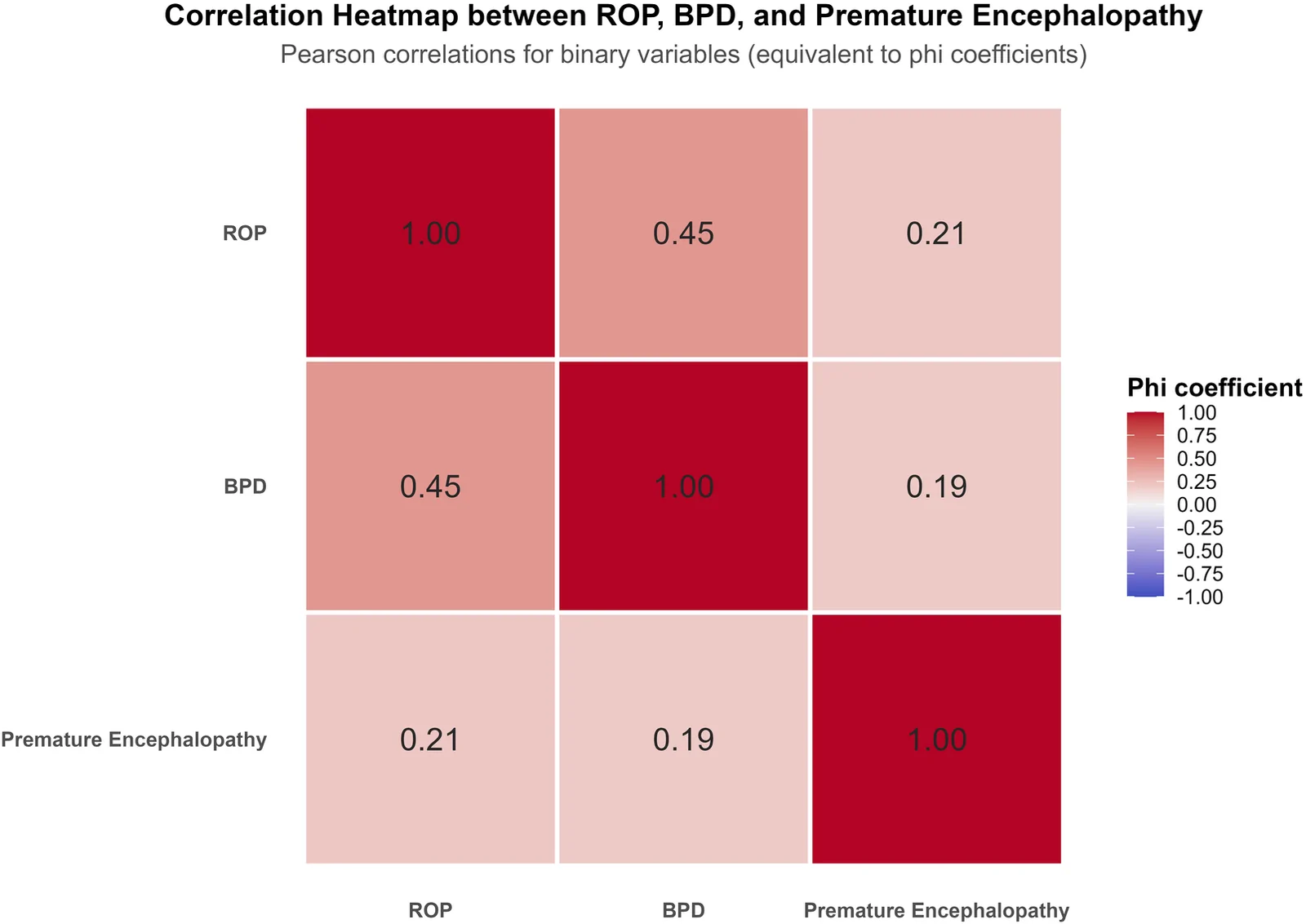
Pairwise associations among retinopathy of prematurity, bronchopulmonary dysplasia, and prematurity-related encephalopathy. The heatmap displays Pearson correlation coefficients for binary variables, which are equivalent to phi coefficients. Coefficients were calculated using available cases for each variable pair. Positive values indicate positive pairwise associations. ROP, retinopathy of prematurity; BPD, bronchopulmonary dysplasia.

In available-case analyses, BPD was substantially more frequent in the ROP group than in the non-ROP group (77/84 [91.7%] vs. 44/86 [51.2%]; prevalence ratio, 1.79; 95% CI, 1.44–2.22; odds ratio, 10.35; 95% CI, 4.15–29.69; Fisher's exact *P* < 0.001). Prematurity-related encephalopathy was likewise more frequent in the ROP group (47/68 [69.1%] vs. 40/83 [48.2%]; prevalence ratio, 1.43; 95% CI, 1.09–1.89; odds ratio, 2.39; 95% CI, 1.17–4.99; Fisher's exact *P* = 0.013). The correlation heatmap (Figure 1) showed positive pairwise associations among ROP, BPD, and prematurity-related encephalopathy. The corresponding raw 2 × 2 contingency tables are presented in Supplementary Supplementary Table S3.

### 3.2 Model comparison

**Figure 2:**
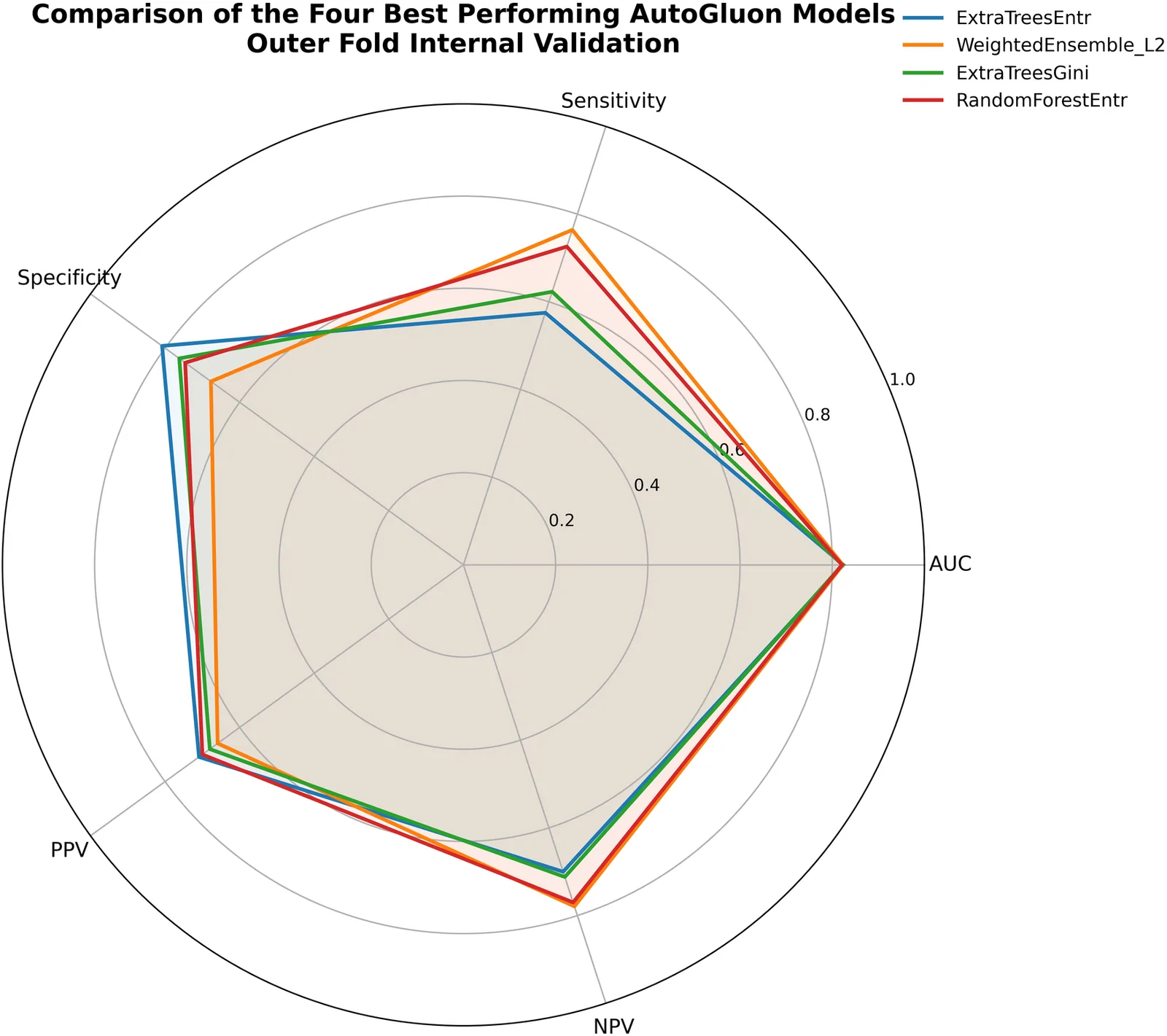
Comparative performance of the four leading AutoGluon models. The radar plot compares the AUC, sensitivity, specificity, positive predictive value, and negative predictive value of ExtraTreesEntr, WeightedEnsemble_L2, ExtraTreesGini, and RandomForestEntr. Metrics were calculated from pooled outer-fold out-of-fold predictions. Higher values indicate better performance. AUC, area under the receiver operating characteristic curve; PPV, positive predictive value; NPV, negative predictive value.

Thirteen individual AutoGluon candidate models and the built-in WeightedEnsemble_L2 were compared using pooled outer-fold out-of-fold predictions (Table 2). AUC values ranged from 0.755 to 0.824. ExtraTreesEntr achieved the numerically highest AUC (0.824), followed closely by WeightedEnsemble_L2 (0.823), ExtraTreesGini (0.822), RandomForestEntr (0.821), and RandomForestGini (0.819). The comparative performance profiles of the four leading models are presented in Figure 2.

**Table 2:** Performance of all AutoGluon models with 95% confidence intervals.

Model	N	AUC (95% CI)	Sensitivity (95% CI)	Specificity (95% CI)	PPV (95% CI)	NPV (95% CI)	F1 (95% CI)
WeightedEnsemble_L2	236	0.823 (0.769–0.873)	0.764 (0.677–0.838)	0.677 (0.595–0.758)	0.659 (0.574–0.742)	0.779 (0.697–0.852)	0.707 (0.634–0.769)
NeuralNetTorch	236	0.802 (0.738–0.858)	0.679 (0.587–0.765)	0.738 (0.662–0.811)	0.679 (0.593–0.763)	0.738 (0.661–0.815)	0.679 (0.601–0.746)
CatBoost	236	0.799 (0.742–0.851)	0.774 (0.690–0.849)	0.692 (0.610–0.773)	0.672 (0.588–0.755)	0.789 (0.711–0.861)	0.719 (0.652–0.782)
KNeighborsUnif	236	0.755 (0.690–0.814)	0.594 (0.500–0.685)	0.831 (0.767–0.894)	0.741 (0.646–0.833)	0.715 (0.640–0.785)	0.660 (0.578–0.734)
LightGBM	236	0.808 (0.751–0.859)	0.670 (0.578–0.755)	0.754 (0.675–0.825)	0.689 (0.598–0.781)	0.737 (0.664–0.810)	0.679 (0.602–0.744)
LightGBMXT	236	0.818 (0.764–0.869)	0.764 (0.680–0.843)	0.685 (0.605–0.763)	0.664 (0.582–0.748)	0.781 (0.702–0.860)	0.711 (0.641–0.776)
NeuralNetFastAI	236	0.813 (0.757–0.865)	0.774 (0.693–0.849)	0.662 (0.580–0.746)	0.651 (0.568–0.733)	0.782 (0.699–0.855)	0.707 (0.637–0.770)
ExtraTreesGini	236	0.822 (0.768–0.873)	0.623 (0.529–0.713)	0.762 (0.686–0.835)	0.680 (0.585–0.774)	0.712 (0.636–0.786)	0.650 (0.568–0.720)
KNeighborsDist	236	0.766 (0.704–0.824)	0.575 (0.478–0.664)	0.738 (0.661–0.812)	0.642 (0.543–0.735)	0.681 (0.600–0.759)	0.607 (0.523–0.680)
XGBoost	236	0.774 (0.711–0.830)	0.726 (0.635–0.813)	0.646 (0.560–0.730)	0.626 (0.539–0.710)	0.743 (0.663–0.822)	0.672 (0.593–0.740)
RandomForestGini	236	0.819 (0.765–0.873)	0.708 (0.619–0.786)	0.762 (0.684–0.836)	0.708 (0.619–0.792)	0.762 (0.689–0.830)	0.708 (0.636–0.770)
RandomForestEntr	236	0.821 (0.768–0.874)	0.726 (0.635–0.811)	0.746 (0.667–0.821)	0.700 (0.612–0.784)	0.770 (0.696–0.842)	0.713 (0.640–0.778)
ExtraTreesEntr	236	0.824 (0.771–0.874)	0.575 (0.475–0.667)	0.808 (0.735–0.873)	0.709 (0.604–0.802)	0.700 (0.623–0.774)	0.635 (0.547–0.709)
LightGBMLarge	236	0.760 (0.694–0.819)	0.632 (0.539–0.721)	0.769 (0.697–0.841)	0.691 (0.595–0.781)	0.719 (0.645–0.793)	0.660 (0.575–0.731)

Values are point estimates with 95% confidence intervals. AUC, area under the receiver operating characteristic curve; PPV, positive predictive value; NPV, negative predictive value; F1, F1 score.

Although ExtraTreesEntr achieved the highest numerical AUC, its sensitivity was only 0.575. WeightedEnsemble_L2 provided comparable discrimination with a higher sensitivity of 0.764 and an NPV of 0.779. NeuralNetFastAI and CatBoost showed the highest sensitivity (both 0.774), whereas KNeighborsUnif achieved the highest specificity (0.831) and PPV (0.741). Because the AUC difference between ExtraTreesEntr and WeightedEnsemble_L2 was only 0.001 and no formal paired comparison was performed, the numerical ranking was not interpreted as evidence of statistical superiority. WeightedEnsemble_L2 was retained because it combined strong discrimination with a more balanced screening-oriented performance profile.

Across the five outer cross-validation folds, eight base learners received nonzero weights in at least one fold-specific WeightedEnsemble_L2 model. After averaging the ensemble weights across all five folds and assigning a weight of zero to models not selected in a given fold, the largest mean weights were observed for LightGBM (20.3%), ExtraTreesGini (18.3%), RandomForestEntr (16.7%), CatBoost (15.0%), and NeuralNetFastAI (13.3%). NeuralNetTorch (7.2%), XGBoost (6.7%), and KNeighborsDist (2.5%) contributed smaller mean weights. Differences in model selection and weighting across folds reflected the fold-specific ensemble-fitting procedure.

The fold-wise imputed WeightedEnsemble_L2 achieved an AUC of 0.823 (95% CI, 0.769–0.873), a PR-AUC of 0.788 (95% CI, 0.706–0.860), and a Brier score of 0.171 (95% CI, 0.147–0.196). Sensitivity was 0.764 (95% CI, 0.677–0.838), specificity was 0.677 (95% CI, 0.595–0.758), PPV was 0.659 (95% CI, 0.574–0.742), and NPV was 0.779 (95% CI, 0.697–0.852). The calibration intercept was −0.095 (95% CI, −0.400 to 0.226), and the calibration slope was 1.046 (95% CI, 0.789–1.392). The pooled confusion matrix comprised 81 true positives, 88 true negatives, 42 false positives, and 25 false negatives.

### 3.3 Model interpretability and translational application

To enable model-specific interpretation, a separate 15-predictor LightGBM model was trained and evaluated using the same fold-wise preprocessing and internal-validation framework. LightGBM was selected because it had the largest mean weight in the cross-validated ensemble and was suitable for TreeSHAP analysis. This standalone model was distinct from the AutoGluon LightGBM candidate reported in Table 2. It achieved an AUC of 0.779 (95% CI, 0.718–0.838), a PR-AUC of 0.736, a Brier score of 0.191, a sensitivity of 0.708, a specificity of 0.700, a PPV of 0.658, an NPV of 0.746, a calibration intercept of −0.122, and a calibration slope of 0.578.

TreeSHAP analyses were performed exclusively on the standalone LightGBM model. The expected model output was 0.465. Global feature importance was summarized using mean absolute SHAP values across all 236 infants, whereas signed SHAP values indicated whether each feature shifted the predicted risk above or below the expected model output.

**Figure 3:**
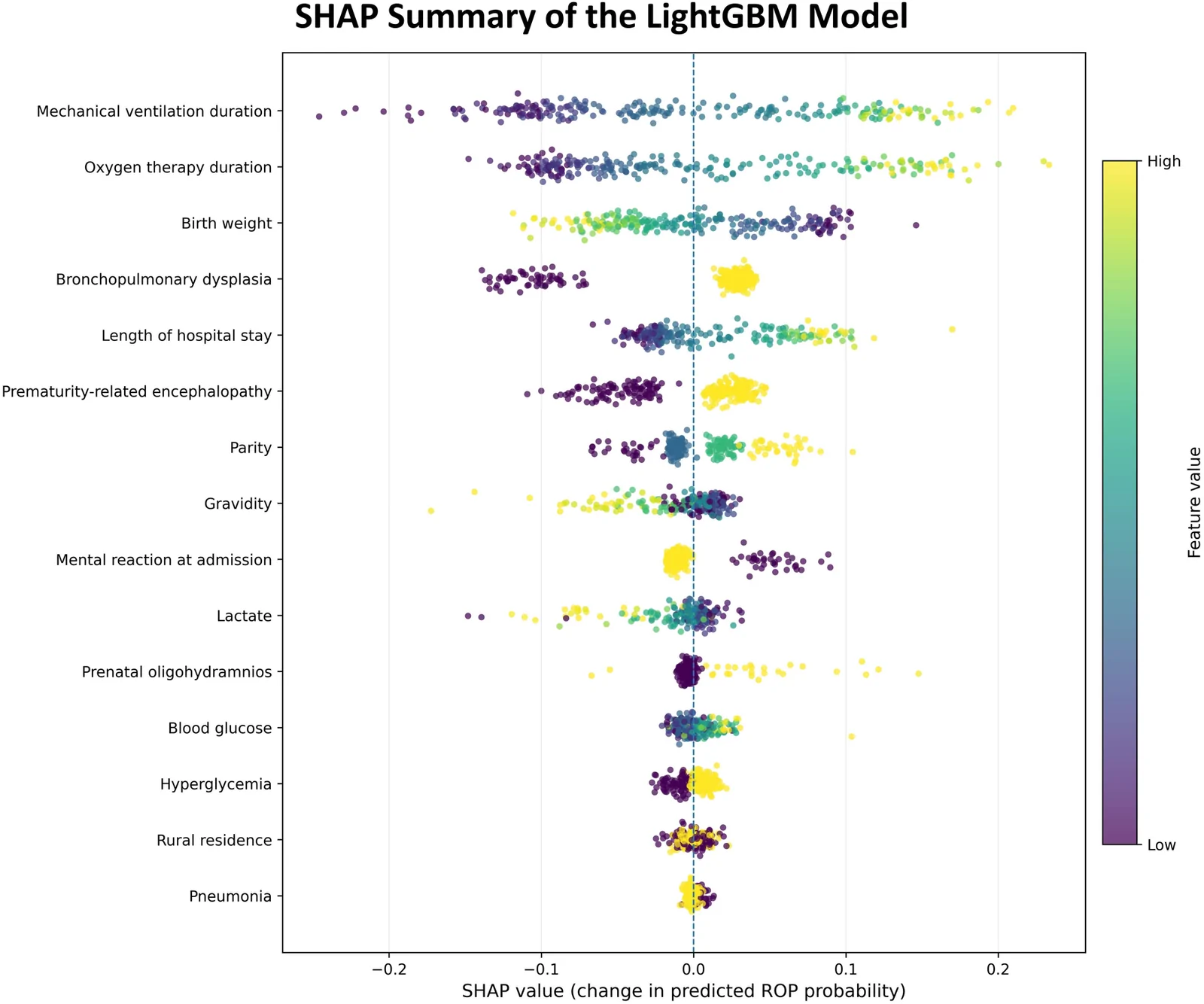
TreeSHAP summary plot for the standalone 15-predictor LightGBM model. Each point represents one infant. The horizontal position indicates the signed SHAP contribution of a predictor to the estimated ROP probability relative to the expected model output of 0.465; positive values shift the prediction toward higher risk, whereas negative values shift it toward lower risk. Colors represent feature values from low to high, and predictors are ordered by their mean absolute SHAP values. The vertical dashed line indicates a SHAP value of zero. SHAP, Shapley additive explanations; ROP, retinopathy of prematurity.

Mechanical ventilation duration had the highest mean absolute SHAP value (0.093), followed by oxygen therapy duration (0.082), birth weight (0.050), bronchopulmonary dysplasia (0.047), length of hospital stay (0.038), and prematurity-related encephalopathy (0.035). The five most influential predictors—mechanical ventilation duration, oxygen therapy duration, birth weight, bronchopulmonary dysplasia, and length of hospital stay—accounted for 68.1% of the total mean absolute SHAP value. Longer mechanical ventilation and oxygen therapy durations generally shifted predictions toward higher risk, whereas higher birth weight shifted predictions toward lower risk. Bronchopulmonary dysplasia and prematurity-related encephalopathy also generally contributed to higher predicted risk. For the infant with the highest predicted risk, the predicted probability was 0.972, compared with an expected model output of 0.465. The largest positive contributions were oxygen therapy duration, mechanical ventilation duration, low birth weight, length of hospital stay, and prematurity-related encephalopathy (Figure 3).

The standalone LightGBM model was implemented within a locally deployed clinical decision-support research prototype. After the relevant information from the index hospitalization has been completed, the 15 clinical variables are entered into the prototype to stratify ROP risk among hospitalized very preterm infants, and the resulting risk estimate and individualized TreeSHAP explanation may help clinicians identify infants who require closer ophthalmic follow-up after discharge. In future applications, the required variables could be automatically extracted from the electronic medical record and transferred to the local prototype, thereby reducing manual entry and standardizing model inputs, while the model itself would remain within the local prototype rather than being directly integrated into the hospital information system. To reduce the risk of data drift, variable definitions, units, coding rules, preprocessing procedures, and model versions should remain standardized and traceable, and input validation should identify missing, implausible, or out-of-range values before model calculation. Predictor distributions, missingness rates, and predicted-risk distributions should be reviewed periodically, and once outcome data become available, discrimination, calibration, and false-negative rates should be reassessed. Any clinically meaningful deterioration should prompt model review and, where appropriate, recalibration or retraining followed by renewed validation. The prototype is intended to complement, rather than replace, established ROP screening recommendations and ophthalmologist assessment. This risk stratification may be particularly valuable before discharge, when clinicians establish individualized ophthalmic follow-up plans based on the infant's overall clinical course and estimated risk.

### 3.4 Analyses of respiratory-support and oxygen-delivery modalities and the treatment-indication subgroup

**Figure 4:**
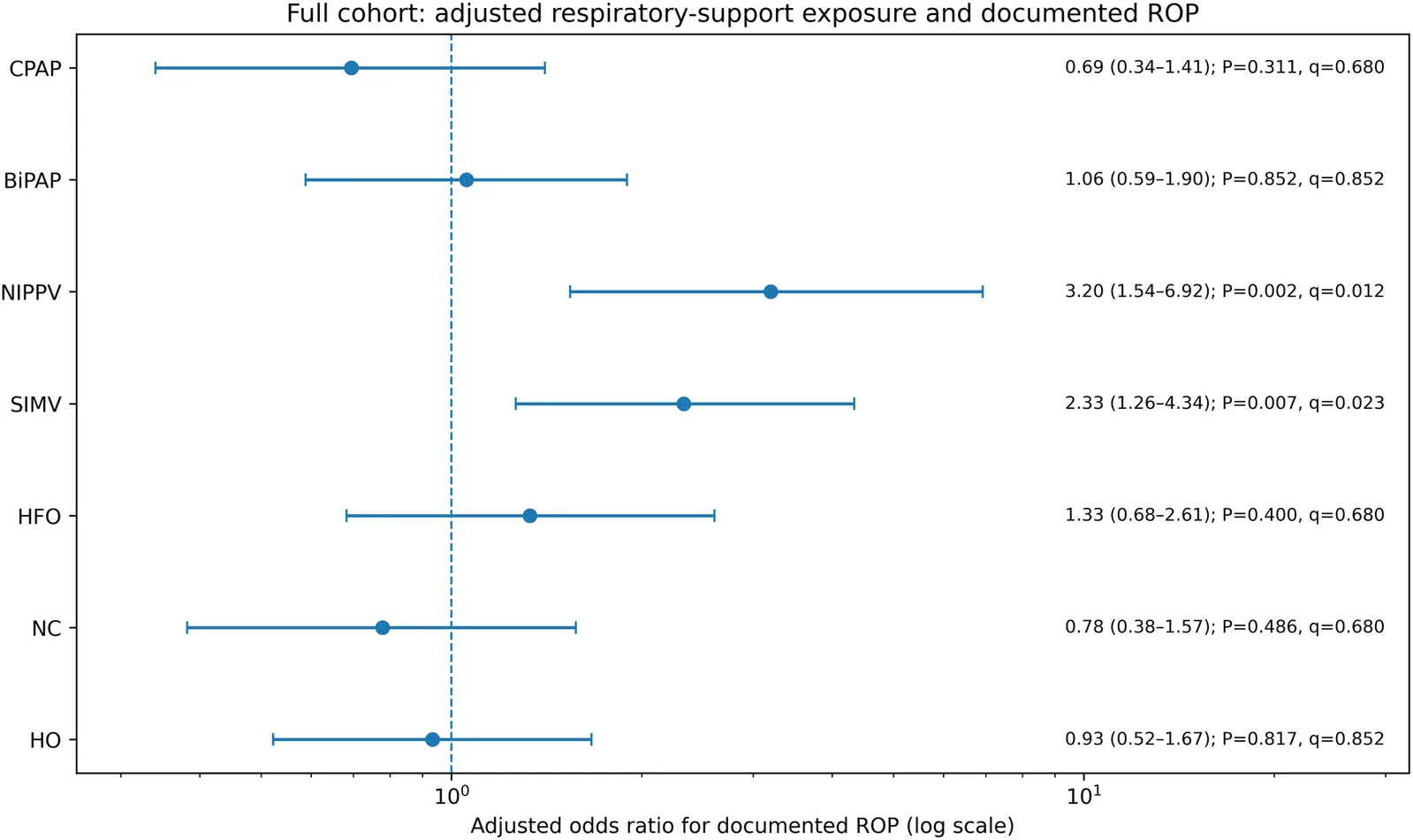
Adjusted Associations of respiratory-support and oxygen-delivery modality exposure with ROP in the full cohort. Points represent adjusted odds ratios, and horizontal lines represent 95% confidence intervals. Models were adjusted for birth weight, cumulative oxygen-therapy duration, and clinical response at NICU admission. The vertical dashed line indicates an odds ratio of 1. *P* values and Benjamini–Hochberg false-discovery-rate-adjusted q values are shown. CPAP, continuous positive airway pressure; BiPAP, bilevel positive airway pressure; NIPPV, nasal intermittent positive-pressure ventilation; SIMV, synchronized intermittent mandatory ventilation; HFO, high-frequency oscillatory ventilation; NC, nasal cannula oxygen; HO, oxygen hood therapy; ROP, retinopathy of prematurity.

In the full cohort, NIPPV and SIMV use were associated with higher odds of ROP after adjustment for birth weight, cumulative oxygen-therapy duration, and clinical response at NICU admission. The adjusted OR was 3.20 for NIPPV (95% CI, 1.54–6.92; *P* = 0.002; FDR-adjusted q = 0.012) and 2.33 for SIMV (95% CI, 1.26–4.34; *P* = 0.007; q = 0.023). Both associations remained significant in sensitivity analyses excluding cumulative oxygen-therapy duration. Among infants who received oxygen hood therapy, each additional 5 days of treatment was associated with higher odds of ROP (adjusted OR, 1.40; 95% CI, 1.10–1.85; *P* = 0.006; q = 0.039). No other modality-specific duration remained significant after false-discovery-rate correction (Figure 4).

**Figure 5:**
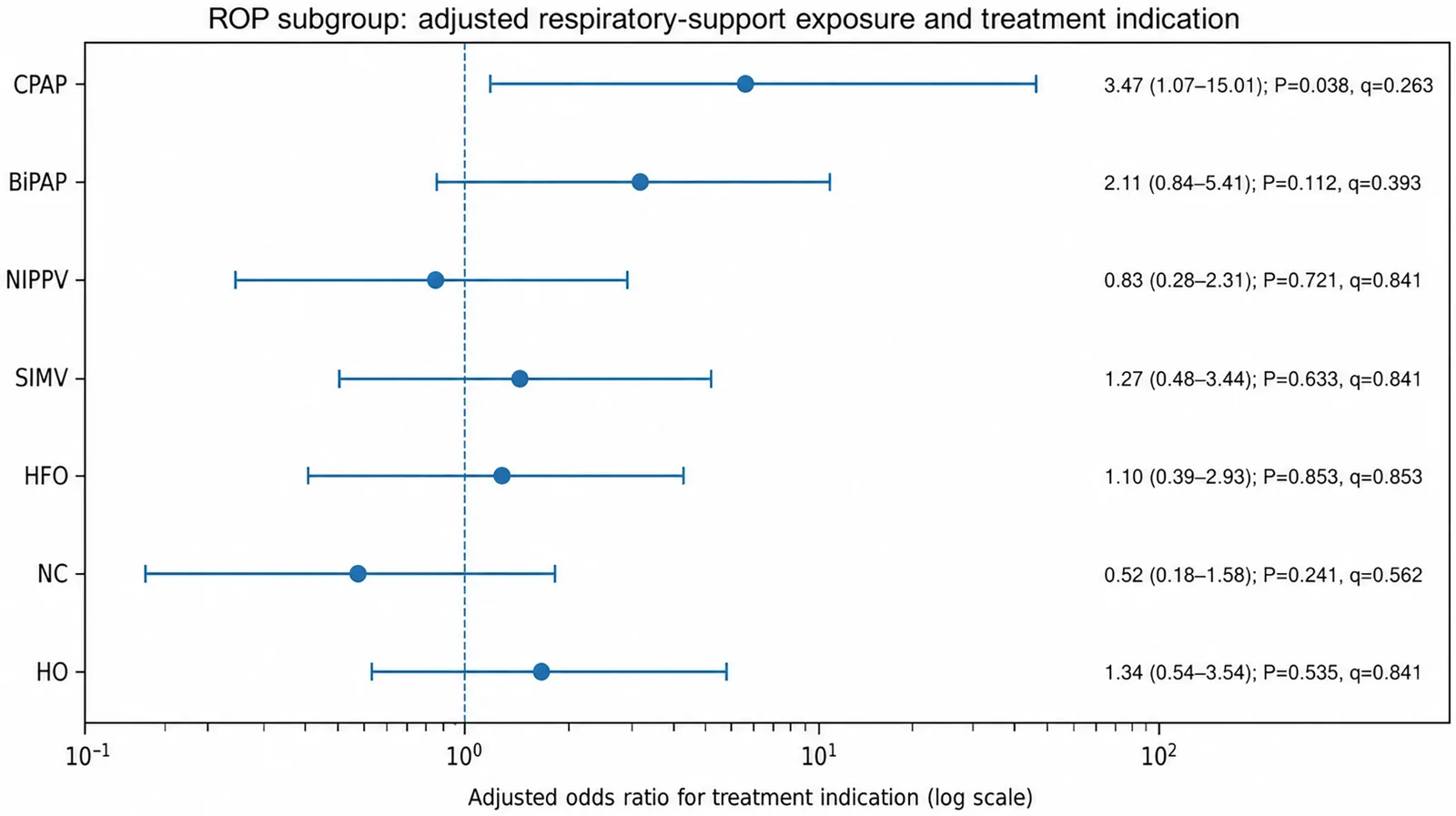
Adjusted associations of respiratory-support and oxygen-delivery modality exposure with treatment indication among infants with ROP. Points represent adjusted odds ratios, and horizontal lines represent 95% confidence intervals. Models were adjusted for birth weight, cumulative oxygen-therapy duration, and clinical response at NICU admission. The vertical dashed line indicates an odds ratio of 1. *P* values and Benjamini–Hochberg false-discovery-rate-adjusted q values are shown. CPAP, continuous positive airway pressure; BiPAP, bilevel positive airway pressure; NIPPV, nasal intermittent positive-pressure ventilation; SIMV, synchronized intermittent mandatory ventilation; HFO, high-frequency oscillatory ventilation; NC, nasal cannula oxygen; HO, oxygen hood therapy; ROP, retinopathy of prematurity.

Within the ROP group, 25 (23.6%) met treatment indications and 81 (76.4%) did not. Compared with infants without treatment indications, those meeting treatment indications had a longer median hospital stay (69 days [IQR, 54–79] vs. 54 days [IQR, 44–68]; *P* = 0.072) and a longer median duration of oxygen therapy (64 days [IQR, 36–75] vs. 49 days [IQR, 34.75–63.25]; *P* = 0.299). Median mechanical ventilation duration was 30 days (IQR, 20–47) and 27 days (IQR, 21.5–40), respectively (*P* = 0.530). Prenatal oligohydramnios was present in 20.0% of infants meeting treatment indications and 9.9% of those without treatment indications (OR, 2.28; 95% CI, 0.67–7.74; *P* = 0.181). None of the 15 clinical-variable comparisons remained statistically significant after false-discovery-rate correction (Figure 5).

Within the ROP group, CPAP use was nominally associated with treatment indication after adjustment for birth weight, cumulative oxygen-therapy duration, and clinical response at NICU admission (adjusted OR, 3.47; 95% CI, 1.07–15.01; *P* = 0.038). However, the association was not significant after false-discovery-rate correction (q = 0.263) and was not reproduced in the sensitivity analysis excluding cumulative oxygen-therapy duration. No other respiratory-support or oxygen-delivery modality, or modality-specific duration, showed a consistent association with treatment indication.

## 4 Discussion

### 4.1 Performance characteristics and complementary value of the modeling framework

WeightedEnsemble_L2 demonstrated a favorable combination of discrimination, classification performance, and calibration. Although its AUC was nearly identical to that of ExtraTreesEntr, the ensemble achieved substantially higher sensitivity while maintaining a favorable NPV. This performance profile is particularly relevant to risk-stratification and screening-oriented applications, in which failure to identify an infant at elevated risk may have greater clinical consequences than additional surveillance of an infant ultimately found not to have ROP. The PR-AUC of 0.788 further indicated effective identification of infants with ROP, while the Brier score of 0.171 reflected reasonable overall accuracy of the predicted probabilities.

The calibration intercept of −0.095 and calibration slope of 1.046 were close to their ideal values of 0 and 1, respectively. These findings suggest that, within the internal-validation setting, the probabilities generated by WeightedEnsemble_L2 were generally aligned with the observed occurrence of ROP and showed limited systematic overestimation, underestimation, or excessive prediction extremity. This is important because a risk-stratification model should provide not only accurate ranking of infants but also probability estimates that meaningfully reflect differences in clinical risk.

The composition of WeightedEnsemble_L2 also illustrates the value of combining complementary modeling strategies. The ensemble incorporated information from boosting, randomized tree, neural-network, and nearest-neighbor learners, with the selected models and their weights varying across cross-validation folds. This heterogeneous structure allowed the ensemble to integrate different representations of nonlinear relationships and interactions among routinely collected clinical variables, thereby reducing dependence on the assumptions or limitations of any single algorithm. The strong performance of several individual models further supports the presence of a reproducible predictive signal within the selected maternal and neonatal variables.

The separately trained LightGBM model complemented the ensemble by providing model-specific interpretation and practical software implementation. Although its predictive performance was lower than that of WeightedEnsemble_L2, it retained meaningful discrimination while enabling TreeSHAP-based global and individual explanations. The use of two models therefore reflects a deliberate division of functions: WeightedEnsemble_L2 was used to characterize the predictive performance achievable through automated ensemble learning, whereas the standalone LightGBM model provided an interpretable and implementable representation of the same 15-variable clinical framework. This complementary design links predictive modeling with transparent interpretation and research-prototype development.

### 4.2 Clinical interpretation of key predictors

In the standalone LightGBM model, mechanical ventilation duration was the most influential predictor, followed by oxygen therapy duration and birth weight. Prolonged respiratory support may reflect pulmonary immaturity, greater illness severity, sustained oxygen exposure, and a more complicated clinical course. In very preterm infants with incompletely developed retinal vasculature, prolonged oxygen exposure, oxygen-saturation fluctuations, and repeated hypoxia–reoxygenation episodes may disrupt normal retinal vascular development and contribute to ROP progression (12, 13). The prominence of respiratory-support variables therefore suggests that ROP risk assessment should incorporate the evolving postnatal clinical course rather than rely exclusively on GA and BW, consistent with previous reviews of postnatal ROP risk factors (14, 15).

Lower birth weight generally shifted LightGBM predictions toward higher ROP risk, consistent with its established role in current screening criteria. Bronchopulmonary dysplasia was also an influential predictor and showed a strong unadjusted association with ROP in the available-case analysis. This relationship may reflect the overlapping effects of pulmonary immaturity, prolonged mechanical ventilation, oxygen exposure, inflammation, and retinal vascular vulnerability, in agreement with previous studies linking BPD and ROP (16–19).

Length of hospital stay and prematurity-related encephalopathy provided additional predictive information. Hospital stay is likely to represent overall illness complexity rather than a directly modifiable risk factor, whereas the contribution of prematurity-related encephalopathy may reflect shared developmental vulnerability and systemic illness. Because mechanical ventilation, oxygen exposure, BPD, pneumonia, and hospital stay describe related aspects of the same clinical course, their SHAP contributions should be interpreted jointly rather than as independent causal effects. Systemic inflammation and hypoxia–reoxygenation injury may also represent shared mechanisms of injury across neonatal organ systems (20, 21).

The first whole-blood glucose concentration on postnatal day 1 was higher in the ROP group than in the non-ROP group (median, 5.45 vs. 4.30 mmol/L; *P* = 0.041). In the univariable modified Poisson regression analysis of 233 infants with available glucose data, each 0.56-mmol/L increase in blood glucose was associated with a 3.0% higher risk of ROP (risk ratio, 1.030; 95% CI, 1.005–1.055; *P* = 0.018). Early glucose elevation may reflect metabolic stress, infection, nutritional support, illness severity, or immature insulin regulation. Previous evidence also suggests that glucose dysregulation may be related to oxidative stress and alterations in IGF-1- and VEGF-related pathways involved in retinal vascular development (22). Lactate was included separately as an indicator of early metabolic and circulatory status and may reflect tissue perfusion, hypoxia, respiratory instability, and overall illness severity.

The modality-specific analyses further showed that NIPPV and SIMV use were associated with higher odds of ROP, while longer oxygen hood therapy was associated with higher odds among exposed infants. These findings should not be interpreted as direct harmful effects of the modalities themselves, because the choice and duration of respiratory support are closely related to respiratory immaturity, illness severity, and cumulative oxygen exposure. Previous studies comparing neonatal ventilation modalities have primarily focused on respiratory outcomes rather than ROP (23, 24). In the ROP subgroup, no respiratory-support or oxygen-delivery modality showed a robust association with treatment indication after false-discovery-rate correction, and the small number of treatment-indicated cases limited the precision of these estimates.

Together, these findings indicate that routinely recorded respiratory support, oxygen exposure, metabolic status, and overall illness burden may provide clinically relevant information for clinical-course-based in-hospital ROP risk stratification.

### 4.3 Treatment-indication subgroup findings

Within the ROP group, those meeting treatment indications tended to have longer hospitalization and oxygen therapy, suggesting a more prolonged and complex clinical course. However, none of the clinical-variable comparisons remained significant after false-discovery-rate correction. The limited number of treatment-indicated cases, together with missing data and the low prevalence of several characteristics, reduced the precision of these estimates. These findings should therefore be considered exploratory and may help inform the selection of candidate predictors for future studies focused specifically on treatment-requiring ROP.

### 4.4 Comparison with existing screening strategies and previous prediction models

Current ROP screening is primarily based on GA and BW and remains essential for identifying infants who require ophthalmic examination. However, these static birth characteristics do not fully reflect the subsequent clinical course of very preterm infants. By integrating 15 routinely collected maternal and neonatal variables, the present framework complements established screening criteria by providing clinical-course-based in-hospital risk stratification rather than replacing guideline-based fundus screening.

Previous ROP prediction studies have used clinical scoring systems, regression models, and individual machine-learning algorithms. AutoGluon enabled multiple fitted models to be compared under the same preprocessing and internal-validation framework and constructed a weighted ensemble from selected learners. The screening score reported by Siswanto et al. achieved AUC values of approximately 0.719–0.732 (25), compared with 0.823 for WeightedEnsemble_L2 in the present cohort, although direct comparison is limited by differences in study populations and validation procedures. Unlike fundus-image-based AI systems, the present framework uses routinely collected NICU clinical data; the two approaches therefore address different but complementary aspects of ROP assessment.

### 4.5 Strengths

This study has several strengths. Model development and evaluation were conducted within a fold-wise internal-validation framework, with imputation, model fitting, and threshold selection restricted to the corresponding development data, thereby reducing information leakage. Thirteen individual AutoGluon models and the built-in WeightedEnsemble_L2 were evaluated using the same data partitions and preprocessing procedures, allowing direct comparison of fitted models. Performance was assessed using discrimination, precision–recall, calibration, classification metrics, Brier score, and bootstrap confidence intervals. In addition, the standalone LightGBM model was evaluated separately and interpreted using model-specific TreeSHAP, ensuring consistency between the reported performance and feature-attribution results. The study also incorporated treatment-indication and respiratory-support analyses, extending the investigation beyond overall ROP classification.

### 4.6 Limitations

This study also has several limitations. Its single-center retrospective design, modest sample size, and absence of external validation may limit generalizability. Several predictors were cumulative measures recorded during hospitalization, and their timing relative to ROP detection was not standardized; therefore, the model should be interpreted as supporting clinical-course-based in-hospital risk stratification rather than real-time dynamic prediction or prediction at a fixed early time point. Missing data and variation in ophthalmic follow-up may also have introduced uncertainty. The treatment-indication subgroup included only 25 infants, and the respiratory-support analyses were exploratory and potentially affected by residual confounding and temporal ambiguity. Finally, SHAP values describe model attribution rather than causal effects, and the standalone LightGBM prototype requires validation of the exact implemented model in external and prospective settings before clinical use.

## 5 Conclusions

This study developed and internally validated an AutoGluon-based framework for clinical-course-based in-hospital ROP risk stratification in very preterm infants. WeightedEnsemble_L2 achieved balanced discrimination and screening-oriented performance, with an AUC of 0.823, while the separately trained LightGBM model enabled TreeSHAP-based interpretation and local research-prototype development. Respiratory support, oxygen exposure, birth weight, bronchopulmonary dysplasia, and prematurity-related encephalopathy provided important information for risk stratification, and exploratory analyses suggested that specific respiratory-support patterns may offer additional clinical information. This framework may complement established GA- and BW-based screening by incorporating the overall clinical course during hospitalization. Multicenter external and prospective validation is required before clinical implementation.

## Data Availability

The original contributions presented in the study are included in the article/Supplementary Material, further inquiries can be directed to the corresponding author.

## Supplementary material

Supplementary Table S1 Data dictionary and definitions of the 15 candidate predictors. The table summarizes the variable labels, clinical definitions, data types, and effect scales used in the regression analyses. Continuous variables were expressed per prespecified unit increase, ordinal variables per one-unit increase, and binary variables as 1 versus 0. BW, birth weight; BPD, bronchopulmonary dysplasia; NICU, neonatal intensive care unit

Supplementary Table S2 Prespecified multiple-imputation settings, implementation details, and distributional diagnostics. The table summarizes the multiple-imputation framework, random seeds, variable constraints, fold-specific preprocessing procedures, and diagnostic comparisons before and after imputation. Ten completed datasets were generated for descriptive analyses, whereas imputation during cross-validation was fitted exclusively within the corresponding training data. The standardized mean shift represents the difference between the pooled completed-data mean and the observed mean divided by the observed-data standard deviation. MICE, multiple imputation by chained equations; ROP, retinopathy of prematurity; BPD, bronchopulmonary dysplasia

Supplementary Table S3 Raw 2 × 2 contingency tables for the associations of ROP with bronchopulmonary dysplasia and prematurity-related encephalopathy. Panel A presents the distribution of bronchopulmonary dysplasia according to ROP group, and Panel B presents the distribution of prematurity-related encephalopathy according to ROP group. Values are based on available cases, and percentages were calculated using the available number of infants within each outcome group. ROP, retinopathy of prematurity; BPD, bronchopulmonary dysplasia

Supplementary Table S4 Definitions for disease-related predictors

## References

[1] Strube YNJ Wright KW. Pathophysiology of retinopathy of prematurity. Saudi J Ophthalmol. (2022) 36(3):239–42. 10.4103/sjopt.sjopt_18_22 36276256 PMC9583358

[2] Kim SJ Port AD Swan R Campbell JP Chan RVP Chiang MF. Retinopathy of prematurity: a review of risk factors and their clinical significance. Surv Ophthalmol. (2018) 63(5):618–37. 10.1016/j.survophthal.2018.04.002 29679617 PMC6089661

[3] de Las Rivas Ramírez N Luque Aranda G Rius Díaz F Pérez Frías FJ Sánchez Tamayo T. Risk factors associated with retinopathy of prematurity development and progression. Sci Rep. (2022) 12(1):21977. 10.1038/s41598-022-26229-4 PMC9767907 36539470

[4] Fierson WM Chiang MF Good W Phelps D Reynolds J Robbins SL. Screening examination of premature infants for retinopathy of prematurity. Pediatrics. (2018) 142(6):e20183061. 10.1542/peds.2018-3061 30478242

[5] Albialy H Rass A. Retinopathy of prematurity: screening and management. Delta J Ophthalmol. (2018) 19(3):205–10. 10.4103/DJO.DJO_4_18

[6] Zhang L Buonfiglio F Fieß A Pfeiffer N Gericke A. Retinopathy of prematurity: targeting hypoxic and redox signaling pathways. Antioxidants. (2024) 13(2):148. 10.3390/antiox13020148 38397746 PMC10885953

[7] Durmaz Engin C Ozturk T Ozkan O Oztas A Selver MA Tuzun F. Prediction of retinopathy of prematurity development and treatment need with machine learning models. BMC Ophthalmol. (2025) 25(1):194. 10.1186/s12886-025-04025-8 40211206 PMC11983953

[8] Jafarizadeh A Maleki SF Pouya P Sobhi N Abdollahi M Pedrammehr S. Current and future roles of artificial intelligence in retinopathy of prematurity. Artif Intell Rev (2025) 58:188. 10.1007/s10462-025-11153-6

[9] Fundus Disease Group of the Chinese Ophthalmological Society, Chinese Medical Association. Chinese Guidelines for screening of retinopathy of prematurity (2014). Zhonghua Yan Ke Za Zhi. (2014) 50(12):933–5. 10.3760/cma.j.issn.0412-4081.2014.12.017 [Article in Chinese].

[10] Chiang MF Quinn GE Fielder AR Ostmo SR Paul Chan RV Berrocal A. International classification of retinopathy of prematurity, third edition. Ophthalmology. (2021) 128(10):e51–68. 10.1016/j.ophtha.2021.05.031 34247850 PMC10979521

[11] Early Treatment For Retinopathy Of Prematurity Cooperative Group. Revised indications for the treatment of retinopathy of prematurity: results of the Early Treatment for Retinopathy of Prematurity randomized trial. Arch Ophthalmol. (2003) 121(12):1684–94. 10.1001/archopht.121.12.1684 14662586

[12] Lin W-C Jordan BK Scottoline B Ostmo SR Coyner AS Singh P. Oxygenation fluctuations associated with severe retinopathy of prematurity. Ophthalmol Sci. (2024) 4(2):100417. 10.1016/j.xops.2023.100417 38059124 PMC10696464

[13] Chen ML Guo L Smith LEH Dammann CEL Dammann O. High or low oxygen saturation and severe retinopathy of prematurity: a meta-analysis. Pediatrics. (2010) 125(6):e1483–92. 10.1542/peds.2009-2218 20498174 PMC4016714

[14] Satnarine T Kin L M C Kevin L. Retinopathy of prematurity: a review of risk factors, oxygen targets, screening criteria. J Med Health Stud. (2022) 3(2):26–36. 10.32996/jmhs.2022.3.2.5

[15] Obolonska OY Vakulenko LI Badogina LP Obolonskyi OI Likhachova IA Kovryga OV. Risk factors for the development of retinopathy in premature infants. Childs Health. (2022) 17(3):138–43. 10.22141/2224-0551.17.3.2022.1509

[16] Wu H Zhang J Zhang J Yu Y Zhang H Han T. Are the risk factors for bronchopulmonary dysplasia and retinopathy of prematurity in very low-birth-weight infants the same? Children. (2025) 12(4):509. 10.3390/children12040509 40310137 PMC12026289

[17] Zhang H Jiang Y Zhang X Su L Yu Y Li L. Risk factors for retinopathy of prematurity among preterm infants with bronchopulmonary dysplasia. J Matern Fetal Neonatal Med. (2025) 38(1):2497058. 10.1080/14767058.2025.2497058 40360447

[18] Podraza W Michalczuk B Jezierska K Domek H Kordek A Łoniewska B. Correlation of retinopathy of prematurity with bronchopulmonary dysplasia. Open Med. (2018) 13(1):67–73. 10.1515/med-2018-0012 PMC5874512 29607416

[19] Buonocore G Perrone S Tataranno M. Oxidative stress and bronchopulmonary dysplasia. J Clin Neonatol. (2012) 1(3):109–14. 10.4103/2249-4847.101683 24027702 PMC3762019

[20] Dammann O Stansfield BK. Neonatal sepsis as a cause of retinopathy of prematurity: an etiological explanation. Prog Retin Eye Res. (2024) 98:101230. 10.1016/j.preteyeres.2023.101230 37984792 PMC10842718

[21] Rognlien AGW Wollen EJ Atneosen-Åsegg M Saugstad OD. Increased expression of inflammatory genes in the neonatal mouse brain after hyperoxic reoxygenation. Pediatr Res. (2014) 77(2):326–33. 10.1038/pr.2014.193 25423075

[22] Cakir B Hellström W Tomita Y Fu Z Liegl R Winberg A. IGF1, serum glucose, and retinopathy of prematurity in extremely preterm infants. JCI Insight. (2020) 5(19):140363. 10.1172/jci.insight.140363 33004691 PMC7566718

[23] Singh SN Malik GK Prashanth GP Singh A Kumar M. High frequency oscillatory ventilation versus synchronized intermittent mandatory ventilation in preterm neonates with hyaline membrane disease: a randomized controlled trial. Indian Pediatr. (2012) 49(5):4. 10.1007/s13312-012-0084-7 22700666

[24] Cools F Henderson-Smart DJ Offringa M Askie LM. Elective high frequency oscillatory ventilation versus conventional ventilation for acute pulmonary dysfunction in preterm infants. Cochrane Database Syst Rev. (2015) 2015(3):CD000104. 10.1002/14651858.CD000104.pub4 25785789 PMC10711725

[25] Siswanto JE Adisasmita AC Ronoatmodjo S Daniswara BA Dijk PH Bos AF. Validation of a screening score model to predict the development of retinopathy of prematurity. Sci Rep. (2025) 15(1):40571. 10.1038/s41598-025-24303-1 41254045 PMC12627429

