# Supplementary material for: AutoGluon-based interpretable machine learning for clinical risk stratification of retinopathy of prematurity in very preterm infants
Source: Front Pediatr. 2026 Sep 4;14:1907714. doi: 10.3389/fped.2026.1907714 (PMC13585999; doi:10.3389/fped.2026.1907714)
Supplement: Supplementary file 1 [file Table1.docx]

**Table 1. Baseline characteristics of the cohort by documented ROP status**

| **Variable** | **Total (n=236)** | **Non-ROP (n=130)** | **ROP (n=106)** | **Statistic** | **P value** |
| --- | --- | --- | --- | --- | --- |
| BW | 1.25±0.24 | 1.34±0.21 | 1.15±0.23 | U=10184.00 | <0.0001 |
| Lac | 2.77±1.88 | 2.91±1.89 | 2.61±1.86 | U=3124.50 | 0.3749 |
| Blood_Glucose | 5.17±2.78 | 4.82±2.56 | 5.61±3.00 | U=5660.50 | 0.0406 |
| Hospital_Days | 49.27±19.03 | 41.47±13.02 | 58.84±20.84 | U=3125.50 | <0.0001 |
| Ventilator_Days | 23.50±14.06 | 17.13±9.51 | 31.39±14.79 | U=2596.50 | <0.0001 |
| Oxygen_Days | 38.55±22.14 | 28.68±15.44 | 50.77±23.13 | U=2901.50 | <0.0001 |
| G | 2.63±1.59 | 2.67±1.71 | 2.58±1.45 | U=6866.00 | 0.9630 |
| P | 1.49±0.97 | 1.44±0.97 | 1.56±0.98 | U=6394.00 | 0.3098 |
| **Rural** |  |  |  | **χ²=0.01** | **0.9097** |
| Rural=1 | 124/235 (52.8%) | 69/129 (53.5%) | 55/106 (51.9%) |  |  |
| Rural=0 | 111/235 (47.2%) | 60/129 (46.5%) | 51/106 (48.1%) |  |  |
| **Hyperglycemia** |  |  |  | **χ²=5.12** | **0.0237** |
| Hyperglycemia=1 | 132/207 (63.8%) | 67/118 (56.8%) | 65/89 (73.0%) |  |  |
| Hyperglycemia=0 | 75/207 (36.2%) | 51/118 (43.2%) | 24/89 (27.0%) |  |  |
| **BPD** |  |  |  | **χ²=32.04** | **<0.0001** |
| BPD=1 | 121/170 (71.2%) | 44/86 (51.2%) | 77/84 (91.7%) |  |  |
| BPD=0 | 49/170 (28.8%) | 42/86 (48.8%) | 7/84 (8.3%) |  |  |
| **Pneumonia** |  |  |  | **χ²=19.21** | **<0.0001** |
| Pneumonia=1 | 137/182 (75.3%) | 56/92 (60.9%) | 81/90 (90.0%) |  |  |
| Pneumonia=0 | 45/182 (24.7%) | 36/92 (39.1%) | 9/90 (10.0%) |  |  |
| **Premature_Encephalopathy** |  |  |  | **χ²=5.87** | **0.0154** |
| Premature_Encephalopathy=1 | 87/151 (57.6%) | 40/83 (48.2%) | 47/68 (69.1%) |  |  |
| Premature_Encephalopathy=0 | 64/151 (42.4%) | 43/83 (51.8%) | 21/68 (30.9%) |  |  |
| **Oligo** |  |  |  | **χ²=0.92** | **0.3384** |
| Oligo=1 | 23/236 (9.7%) | 10/130 (7.7%) | 13/106 (12.3%) |  |  |
| Oligo=0 | 213/236 (90.3%) | 120/130 (92.3%) | 93/106 (87.7%) |  |  |
| **Reaction** |  |  |  | **χ²=0.19** | **0.6618** |
| Reaction=1 | 193/231 (83.5%) | 107/126 (84.9%) | 86/105 (81.9%) |  |  |
| Reaction=0 | 38/231 (16.5%) | 19/126 (15.1%) | 19/105 (18.1%) |  |  |

Data are presented as mean±SD for continuous and ordinal variables and as n/N (%) for categorical variables.

Continuous and ordinal variables were compared using two-sided Mann–Whitney U tests. Binary variables were compared using Pearson χ² tests with Yates continuity correction.
