# Supplementary material for: AutoGluon-based interpretable machine learning for clinical risk stratification of retinopathy of prematurity in very preterm infants
Source: Front Pediatr. 2026 Sep 4;14:1907714. doi: 10.3389/fped.2026.1907714 (PMC13585999; doi:10.3389/fped.2026.1907714)
Supplement: Supplementary file 2 [file Table2.docx]

**Table 2 Performance of all AutoGluon models with 95% confidence intervals**

| **Model** | **N** | **AUC (95% CI)** | **Sensitivity (95% CI)** | **Specificity (95% CI)** | **PPV (95% CI)** | **NPV (95% CI)** | **F1 (95% CI)** |
| --- | --- | --- | --- | --- | --- | --- | --- |
| WeightedEnsemble_L2 | 236 | 0.823 (0.769–0.873) | 0.764 (0.677–0.838) | 0.677 (0.595–0.758) | 0.659 (0.574–0.742) | 0.779 (0.697–0.852) | 0.707 (0.634–0.769) |
| NeuralNetTorch | 236 | 0.802 (0.738–0.858) | 0.679 (0.587–0.765) | 0.738 (0.662–0.811) | 0.679 (0.593–0.763) | 0.738 (0.661–0.815) | 0.679 (0.601–0.746) |
| CatBoost | 236 | 0.799 (0.742–0.851) | 0.774 (0.690–0.849) | 0.692 (0.610–0.773) | 0.672 (0.588–0.755) | 0.789 (0.711–0.861) | 0.719 (0.652–0.782) |
| KNeighborsUnif | 236 | 0.755 (0.690–0.814) | 0.594 (0.500–0.685) | 0.831 (0.767–0.894) | 0.741 (0.646–0.833) | 0.715 (0.640–0.785) | 0.660 (0.578–0.734) |
| LightGBM | 236 | 0.808 (0.751–0.859) | 0.670 (0.578–0.755) | 0.754 (0.675–0.825) | 0.689 (0.598–0.781) | 0.737 (0.664–0.810) | 0.679 (0.602–0.744) |
| LightGBMXT | 236 | 0.818 (0.764–0.869) | 0.764 (0.680–0.843) | 0.685 (0.605–0.763) | 0.664 (0.582–0.748) | 0.781 (0.702–0.860) | 0.711 (0.641–0.776) |
| NeuralNetFastAI | 236 | 0.813 (0.757–0.865) | 0.774 (0.693–0.849) | 0.662 (0.580–0.746) | 0.651 (0.568–0.733) | 0.782 (0.699–0.855) | 0.707 (0.637–0.770) |
| ExtraTreesGini | 236 | 0.822 (0.768–0.873) | 0.623 (0.529–0.713) | 0.762 (0.686–0.835) | 0.680 (0.585–0.774) | 0.712 (0.636–0.786) | 0.650 (0.568–0.720) |
| KNeighborsDist | 236 | 0.766 (0.704–0.824) | 0.575 (0.478–0.664) | 0.738 (0.661–0.812) | 0.642 (0.543–0.735) | 0.681 (0.600–0.759) | 0.607 (0.523–0.680) |
| XGBoost | 236 | 0.774 (0.711–0.830) | 0.726 (0.635–0.813) | 0.646 (0.560–0.730) | 0.626 (0.539–0.710) | 0.743 (0.663–0.822) | 0.672 (0.593–0.740) |
| RandomForestGini | 236 | 0.819 (0.765–0.873) | 0.708 (0.619–0.786) | 0.762 (0.684–0.836) | 0.708 (0.619–0.792) | 0.762 (0.689–0.830) | 0.708 (0.636–0.770) |
| RandomForestEntr | 236 | 0.821 (0.768–0.874) | 0.726 (0.635–0.811) | 0.746 (0.667–0.821) | 0.700 (0.612–0.784) | 0.770 (0.696–0.842) | 0.713 (0.640–0.778) |
| ExtraTreesEntr | 236 | 0.824 (0.771–0.874) | 0.575 (0.475–0.667) | 0.808 (0.735–0.873) | 0.709 (0.604–0.802) | 0.700 (0.623–0.774) | 0.635 (0.547–0.709) |
| LightGBMLarge | 236 | 0.760 (0.694–0.819) | 0.632 (0.539–0.721) | 0.769 (0.697–0.841) | 0.691 (0.595–0.781) | 0.719 (0.645–0.793) | 0.660 (0.575–0.731) |

Values are point estimates with 95% confidence intervals. AUC, area under the receiver operating characteristic curve; PPV, positive predictive value; NPV, negative predictive value; F1, F1 score.
