# Supplementary material for: AutoGluon-based interpretable machine learning for clinical risk stratification of retinopathy of prematurity in very preterm infants
Source: Front Pediatr. 2026 Sep 4;14:1907714. doi: 10.3389/fped.2026.1907714 (PMC13585999; doi:10.3389/fped.2026.1907714)
Supplement: Supplementary file 3 [file Table3.docx]

**Supplementary Table S3. Raw 2 × 2 contingency tables for the associations of ROP with bronchopulmonary dysplasia and prematurity-related encephalopathy**

**Panel A. ROP and bronchopulmonary dysplasia**

| **Outcome group** | **BPD present, n** | **BPD absent, n** | **Available n** | **Present, %** |
| --- | --- | --- | --- | --- |
| ROP group | 77 | 7 | 84 | 91.7 |
| Non-ROP group | 44 | 42 | 86 | 51.2 |
| Total | 121 | 49 | 170 | 71.2 |

**Panel B. ROP and prematurity-related encephalopathy**

| **Outcome group** | **Prematurity-related encephalopathy present, n** | **Prematurity-related encephalopathy absent, n** | **Available n** | **Present, %** |
| --- | --- | --- | --- | --- |
| ROP group | 47 | 21 | 68 | 69.1 |
| Non-ROP group | 40 | 43 | 83 | 48.2 |
| Total | 87 | 64 | 151 | 57.6 |

Values are raw available-case counts. ROP indicates retinopathy of prematurity documented during hospitalization; BPD, bronchopulmonary dysplasia. Percentages were calculated using the available n within each outcome group.
