# Supplementary material for: AutoGluon-based interpretable machine learning for clinical risk stratification of retinopathy of prematurity in very preterm infants
Source: Front Pediatr. 2026 Sep 4;14:1907714. doi: 10.3389/fped.2026.1907714 (PMC13585999; doi:10.3389/fped.2026.1907714)
Supplement: Supplementary file 4 [file Table4.docx]

**Supplementary Table S4. Definitions for disease-related predictors**

| **Variable** | **Operational definition** | **Reference standard** |
| --- | --- | --- |
| Hyperglycemia | Neonatal hyperglycemia was defined as at least two peripheral whole-blood glucose measurements >7.0 mmol/L (125 mg/dL) within 12 hours during NICU hospitalization. | Chinese neonatal clinical definition commonly used with reference to Practical Neonatology, 5th edition. |
| Bronchopulmonary dysplasia (BPD) | Bronchopulmonary dysplasia was defined according to the 2001 NICHD consensus criteria. For infants born at <32 weeks' gestation, BPD was diagnosed when supplemental oxygen (FiO₂ >21%) was required for at least 28 cumulative days. Severity was assessed at 36 weeks' postmenstrual age or at discharge, whichever occurred first: mild BPD, breathing room air; moderate BPD, requiring FiO₂ <30%; and severe BPD, requiring FiO₂ ≥30% and/or positive-pressure respiratory support. For the binary variable used in this study, mild, moderate, or severe BPD was coded as BPD present. | 2001 NICHD consensus definition; Chinese expert consensus on the clinical management of BPD in preterm infants (2020). |
| Neonatal pneumonia | Neonatal infectious pneumonia documented by the attending neonatologist on the basis of compatible respiratory and/or systemic clinical manifestations together with supportive chest imaging findings, with laboratory or microbiological evidence considered when available and noninfectious neonatal respiratory disorders excluded. | Chinese guideline for neonatal infectious pneumonia (2025). |
| Prematurity-related encephalopathy | A clinician-documented diagnosis of brain injury associated with prematurity, based on neurological abnormalities and/or cranial ultrasonography or magnetic resonance imaging findings, including cerebral white-matter injury or periventricular leukomalacia, germinal matrix-intraventricular hemorrhage, periventricular hemorrhagic infarction, cerebellar hemorrhage, or other acquired preterm brain injury. Because no universally accepted binary diagnostic criterion exists, classification followed the diagnosis recorded in the NICU medical record. | Concept of encephalopathy of prematurity described by Volpe; neuroimaging findings interpreted from routine neonatal care. |
| Prenatal oligohydramnios | Oligohydramnios documented in the obstetric medical record on prenatal ultrasonography, defined as a single deepest vertical pocket <2 cm. When the amniotic fluid index was used in the source record, an amniotic fluid index ≤5 cm was considered oligohydramnios. | ACOG guidance for oligohydramnios. |
